# Abundance and Distribution of Enteric Bacteria and Viruses in Coastal and Estuarine Sediments—a Review

**DOI:** 10.3389/fmicb.2016.01692

**Published:** 2016-11-01

**Authors:** Francis Hassard, Ceri L. Gwyther, Kata Farkas, Anthony Andrews, Vera Jones, Brian Cox, Howard Brett, Davey L. Jones, James E. McDonald, Shelagh K. Malham

**Affiliations:** ^1^School of Ocean Sciences, Bangor UniversityBangor, UK; ^2^Department of Engineering and Innovation, Open UniversityMilton Keynes, UK; ^3^School of Environment, Natural Resources and Geography, Bangor UniversityBangor, UK; ^4^UK Water Industry Research LimitedLondon, UK; ^5^Atkins LimitedBristol, UK; ^6^Atkins LimitedWarrington, UK; ^7^Thames Water UtilitiesReading, UK; ^8^School of Biological Sciences, Bangor UniversityBangor, UK

**Keywords:** sediment, viable but non-culturable bacteria, biofilm, fecal indicator organisms, resuspension, survival, virus

## Abstract

The long term survival of fecal indicator organisms (FIOs) and human pathogenic microorganisms in sediments is important from a water quality, human health and ecological perspective. Typically, both bacteria and viruses strongly associate with particulate matter present in freshwater, estuarine and marine environments. This association tends to be stronger in finer textured sediments and is strongly influenced by the type and quantity of clay minerals and organic matter present. Binding to particle surfaces promotes the persistence of bacteria in the environment by offering physical and chemical protection from biotic and abiotic stresses. How bacterial and viral viability and pathogenicity is influenced by surface attachment requires further study. Typically, long-term association with surfaces including sediments induces bacteria to enter a viable-but-non-culturable (VBNC) state. Inherent methodological challenges of quantifying VBNC bacteria may lead to the frequent under-reporting of their abundance in sediments. The implications of this in a quantitative risk assessment context remain unclear. Similarly, sediments can harbor significant amounts of enteric viruses, however, the factors regulating their persistence remains poorly understood. Quantification of viruses in sediment remains problematic due to our poor ability to recover intact viral particles from sediment surfaces (typically <10%), our inability to distinguish between infective and damaged (non-infective) viral particles, aggregation of viral particles, and inhibition during qPCR. This suggests that the true viral titre in sediments may be being vastly underestimated. In turn, this is limiting our ability to understand the fate and transport of viruses in sediments. Model systems (e.g., human cell culture) are also lacking for some key viruses, preventing our ability to evaluate the infectivity of viruses recovered from sediments (e.g., norovirus). The release of particle-bound bacteria and viruses into the water column during sediment resuspension also represents a risk to water quality. In conclusion, our poor process level understanding of viral/bacterial-sediment interactions combined with methodological challenges is limiting the accurate source apportionment and quantitative microbial risk assessment for pathogenic organisms associated with sediments in aquatic environments.

## Introduction

There are a multitude of bacteria and viruses naturally present within the aquatic environment of which the vast majority are not derived from humans (Rosenwasser et al., 2016). In addition, the majority of bacteria and viruses derived from humans are benign from a human health perspective, however, a small component is considered pathogenic (Filippini and Middelboe, 2007; Lowther et al., 2012). Human pathogenic bacteria and viruses released from point (e.g., wastewater treatment plants) and diffuse sources (e.g., agricultural land) frequently contaminate water bodies downstream and therefore represent a potential risk to human health (e.g., during recreation, contamination of food and drinking water). A large proportion of the pathogenic organisms present in water may also become associated with the sediment, which can be subject to resuspension (Davies et al., 1995; Anderson et al., 2005; Drummond et al., 2014a) and could represent a significant mechanism for delivering pathogens to coastal waters (Yamahara et al., 2007). In coastal and estuarine environments, the survival of fecal indicator organisms (FIOs; indicating the potential presence of pathogenic bacteria and viruses) is positively linked to the concentration of suspended matter in the water column (Howell et al., 1996). In contrast, other factors such as elevated temperatures and exposure to UV radiation tend to have a negative effect on microbial survival in the water column (Chigbu et al., 2005; Kay et al., 2005). Viruses have also been shown to readily adsorb to solid matter in the water (reviewed in Jin and Flury, 2002). Viral particles associated with solids may travel long distances in water, or settle out during transit, where they become more concentrated in the sediments that in the overlying water column. Viral attachment to solid particles may result in permanent inactivation of the virus, however the adsorbed virus particles are often protected from inactivation from stressors (e.g., UV) by the surface they are attached to Schijven and Hassanizadeh (2000) and Chrysikopoulos and Aravantinou (2012). Current monitoring schemes, and the majority of research in this field, typically focuses on quantifying fecally derived bacteria and viruses in the water column, however, numbers within the sediment (including beaches, riverbanks and mudflats) are often orders of magnitude higher (Rao et al., 1986a; Duhamel and Jacquet, 2006; Vignaroli et al., 2013, 2015; Perkins et al., 2014). Greater abundance of pathogenic organisms and viruses in the sediment reservoir is linked to their binding to particle surfaces and enhanced survival within the biofilm matrix (Smith et al., 1978; LaBelle and Gerba, 1980; Danovaro et al., 2008; Pachepsky and Shelton, 2011). Sediments therefore act as a potential reservoir of pathogens and FIOs in aquatic environments that remain undetected until they re-enter the water column due to the action of rainfall, wind, waves, tides recreational boats, and dredging (Howell et al., 1996; Jamieson R. C. et al., 2005) or are accumulated by filter-feeding shellfish destined for human consumption (Lowther et al., 2012; Malham et al., 2014). The survival of human pathogenic bacteria and viruses in rivers and the marine environment is highly species and strain specific (Gerba et al., 1980; Anderson et al., 2005; Byappanahalli et al., 2006). This makes it difficult to generalize about the behavior of pathogenic organisms in a risk assessment context, particularly when they may come from sources which vary both spatially and temporally. Further, these pathogens may have a markedly different viability in aquatic ecosystems compared to non-pathogenic indicator organisms that are frequently used to represent fecal pollution in environmental monitoring (Sinton et al., 2002). Due to the emergence of new pathogens and the need to reduce the economic and social burden of human disease outbreaks, the source-apportionment and transmission of many disease-causing agents is receiving increased attention (Dobrindt et al., 2004; Vignaroli et al., 2013). Within this, there is a clear need to improve our understanding of the behavior, fate and potential mitigation of pathogens associated with sediments as well as the main water body itself. Adequate consideration of pathogens in sediments will enhance our ability to achieve regulatory compliance with legislation associated with protecting bathing and shellfish waters and in the provision of more robust risk assessments (Danovaro et al., 2008; Pachepsky and Shelton, 2011; Malham et al., 2014). Despite significant investment and research into the factors governing bacterial and viral association with sediments, areas such as the factors governing bacterial resuscitation from a viable but non-culturable (VBNC) state, viral quantification in sediments, and resuspension requires additional attention.

## Abundance and distribution of fecally derived bacteria and viruses

To effectively determine the human health risk associated with coastal and estuarine sediments, it is important to quantify the size of the pathogen pool. The abundance of FIOs such as *Escherichia coli* and *Enterococcus* spp. has been well studied, however, further attention is required for pathogens such as *Campylobacter* spp., *Salmonella* spp., *E. coli* O157:H7 and norovirus, which may cause illness through shellfish consumption or exposure to recreational water (Malham et al., 2014). Previous research has primarily focused on the presence/absence of these microorganisms in sediments, but for an apportionment of risk, a quantitative approach is required (Ramaiah et al., 2005; Setti et al., 2009; Carr et al., 2010; Soares de Lima Grisi and Gorlach-Lira, 2010). The reported number of fecally associated bacteria in coastal and estuarine environment is typically between 0 and 10^4^ colony forming units (CFU) or most probable number (MPN)/100 ml for water and 10^1^ to 10^6^ CFU or MPN/100 g wet weight for sediment (Table 1). Similar trends have been observed in viral abundance in marine and estuarine sediment (Table 1), however, the relative difference in water/sediment abundance cannot be assessed due to the small sample size. Nonetheless, Staggemeier et al. (2015a,b) directly compared the concentrations of adenoviruses in corresponding water and sediment samples derived from freshwater streams, dams, and springs and found that the viral abundance in sediment was significantly higher than in the overlying water. Importantly, they found that adenoviruses may be present in sediment in the absence of the virus in the water column. Anderson et al. (2005) found that sediment had greater spatial variability in bacterial abundance than water, and that populations of enteric organisms can persist in the environment. The high natural variability in the sediment fraction for both bacteria and viruses, has been linked to methodological differences in dissociation from sediment particles which may result in inconsistent enumeration (Anderson et al., 2005; Miura et al., 2011; Pachepsky and Shelton, 2011).

**Table 1:** **Abundance of fecal bacteria and viruses associated with coastal and estuarine sediments**.

**Bacteria**	**Sediments Range or average**	**Water column Range or average**	**References**
	**CFU or MPN 100 g WW^−1^**	**CFU or MPN 100 ml^−1^**	
Fecal coliforms	80–200,000	8–9400	Alcântara and Almeida, 1995; Lucena et al., 1996; Bonilla et al., 2007; Abdelzaher et al., 2010; Luna et al., 2010; Vignaroli et al., 2013; Borade et al., 2014.
*E. coli*	19–100,000	0–6700	Evanson and Ambrose, 2006; Bonilla et al., 2007; Abdelzaher et al., 2010; Stumpf et al., 2010; Borade et al., 2014.
Fecal Streptococci	190–19,000	6–240	Alcântara and Almeida, 1995; Lucena et al., 1996.
*Enterococcus* spp.	80–136,000	0–240	Evanson and Ambrose, 2006; Bonilla et al., 2007; Abdelzaher et al., 2010; Stumpf et al., 2010; Vignaroli et al., 2013.
*E. faecalis*	ND	200	Borade et al., 2014.
*Clostridium perfringens*	300–1,500,000	<2–13	Lucena et al., 1996; Abdelzaher et al., 2010.
*Staphylococcus aureus*	ND	ND	Abdelzaher et al., 2010.
*Salmonella* spp.	ND–262,500	600–1500	Borade et al., 2014.
*Shigella* spp.	ND	1600	Borade et al., 2014.
*Proteus* spp. and *Klebsiella* spp.	6300–543,700	5400–5600	Borade et al., 2014.
*Aeromonas* spp.	36,000	–	Lucena et al., 1996.
*Vibrio* spp.^*^	31,300–756,200	3000–6600	Borade et al., 2014.
**Viruses**	**Sediments Range or average**	**Water column Range or average**	**References**
	**PFU or GC 100 g WW^−1^**	**PFU or GC 100 ml^−1^**	
Enterovirus	3.3–19.08 (6–75)	ND–160 (ND)	Gerba et al., 1977a; Rao et al., 1984, 1986a; Le Guyader et al., 1994; Alcântara and Almeida, 1995; Lucena et al., 1996; Green and Lewis, 1999; Abdelzaher et al., 2010; Miura et al., 2011.^a^
Norovirus	ND	ND	Abdelzaher et al., 2010.
Norovirus GI	ND (24)	ND (0)	Miura et al., 2011; Norman et al., 2013.^a^
Norovirus GII	BDL (ND–6)	ND	Miura et al., 2011; Norman et al., 2013.
Rotavirus	12/4	31–265	Rao et al., 1986a; Alcântara and Almeida, 1995; Green and Lewis, 1999.
Hepatitis A virus	(0–87.5)	(0)	Le Guyader et al., 1994; Green and Lewis, 1999; Abdelzaher et al., 2010.^a^
Human adenovirus	197,000–6,960,000	15,700–20,800,000	Staggemeier et al., 2015a^**^
Human polyomavirus	(Present)	(Present)	Abdelzaher et al., 2010.^a^
Somatic coliphage	ND–240,000 (36)	<1–6 (19)	Alcântara and Almeida, 1995; Lucena et al., 1996; Bonilla et al., 2007.^a^
F+ coliphage	ND–102 (2)	<1–3 (0)	Alcântara and Almeida, 1995; Bonilla et al., 2007.^a^
FRNA-bacteriophage	ND–20	<1–3	Alcântara and Almeida, 1995.
*Bacteroides fragilis* bacteriophage	0–2400	0–2640	Alcântara and Almeida, 1995; Lucena et al., 1996.

* *Vibrio spp. are ubiquitous in the marine environment and facultative pathogens*.

** *Human adenovirus detected in freshwater. ND, none detected. –, not analysed; CFU, Colony forming units; MPN, Most probable number; WW, Wet weight. FRNA bacteriophage, Male specific (F) RNA bacteriophage*.

a *Numbers in parenthesis indicate the prevalence of the virus in a separate study (%) BDL, below detection limit (qPCR); PFU, Plaque forming units; GC, Gene Copies*.

Pathogens and FIOs also associate with suspended solids (flocs) present in the overlying water column (Rao et al., 1984, 1986a; Jamieson R. et al., 2005). The floc fraction is prone to resuspend easily (Pachepsky et al., 2009a) and is an important but poorly quantified contributor to bacterial loading for water quality monitoring (Malham et al., 2014). However, flocs are ephemeral and prone to break up on disturbance, which provides a technical challenge to enumeration. Numerous studies, have reported a decrease in the number of bacteria and viruses with sediment depth (Obiri-Danso and Jones, 2000; Filippini and Middelboe, 2007; Berthe et al., 2008). Recent research showed a two-log reduction in culturable *E. coli* from the sediment surface (top 1 cm) to 4 cm in depth (Pachepsky and Shelton, 2011). Generally, the top 2 cm of sediment is considered to have high FIO abundance whereas below 2 cm has significantly lower abundance (Ferguson et al., 1996; Haller and Amedegnato, 2009; Drummond et al., 2014a). Distinct seasonality of bacteria in sediments has been observed, with greater abundance in autumn-winter months compared to spring-summer months (Goyal et al., 1977; Crabill et al., 1999). In contrast, Ishii et al. (2006) found that summer to autumn had greater abundance in soils and winter to spring had the lowest abundance. Meays et al. (2006) noted a distinct diurnal pattern in *E. coli* abundance in the water column, possibly due to UV light inactivation (Kay et al., 2005; Walters et al., 2013), while the greater stability and protection from stressful conditions could reduce short term changes in abundance. Physio-chemical conditions such as temperature, turbidity, salinity, nutrient and oxygen concentrations and water depth are all important factors controlling the distribution of bacteria (Perkins et al., 2014). The weather, season, disease prevalence in the community; tides and freshwater inputs; time of day; sediment type (sand/mud) and deposition rates; distance from the shore; and predation by, and competition with, the intrinsic microbial community also affects the abundance and distribution of bacteria and viruses (Kirschner et al., 2004; Jamieson R. C. et al., 2005). The complexity of interacting factors that influence pathogen and FIO survival in sediments often restricts direct comparison between studies. Effective surveillance alongside sufficient site/sediment characterization may enable further insights into the influence of the sediment fraction on bathing water quality (Ouattara et al., 2013; Huang et al., 2015). Reports suggest that the number of infectious or culturable pathogens may correlate poorly with the number detected by molecular approaches. Therefore, integrated surveillance schemes using both molecular detection of bacterial/viral genomes by PCR and culture-based methods (e.g., bacterial culture or viral infectivity cell culture tests) may be required (Bae and Schwab, 2008). However, high degrees of inhibition at either the extraction or genome quantification stages suggest that optimization and standardization of molecular methodology in sediments is also required (Miura et al., 2011).

Enteric phages (e.g., F^+^ RNA coliphages) have been utilized as general markers of fecal pollution. Advantages of this approach includes, target specificity (each phage is typically specific to one host) and their greater environmental persistence in comparison to FIOs; typically 3-fold longer under controlled conditions (Allwood et al., 2003). In addition, source apportionment can be undertaken using different genogroups of F^+^ RNA coliphages (e.g., I, IV for animal and II and III for human) (Shahrampour et al., 2015). Concentrations of F^+^ RNA coliphages were between 9 and 20 fold higher in sediments than the overlying water column (Alcântara and Almeida, 1995). Under controlled conditions, F^+^ RNA coliphages show poor correlation with *E. coli*, therefore cannot be readily compared to larger historic datasets (usually *E. coli* or intestinal enterococcus). However, coliphages correlate better with disease incidence and concentrations of pathogens (e.g., norovirus; Doré et al., 2000). Typically, next generation approaches are being used for microbial source tracking (See Section Outlook), however, F^+^ RNA coliphages still provide a useful indicator of viral culturability.

## Sediment characteristics governing bacteria particle interaction

### Bacterial adsorption

Bacterial adsorption principally occurs through physicochemical forcing as described by the extended Derjaguin–Landau–Verwey–Overbeek (DVLO) theory (van Loosdrecht et al., 1989). However, a number of factors can increase the rate of association with particle surfaces. Hermansson (1999) showed that a high ionic strength promotes adsorption between particulate matter and bacteria (Jiang et al., 2007). Cao et al. (2011) found that bacterial adsorption to extracellular polymeric substance (EPS) occurs at a greater rate in the presence of sodium ions. Cations reduce the repulsive electrostatic charge of clay particles and allow formation of cation bridges between functional groups of EPS and negatively charged sites of clays. After the long range DVLO interactions have occurred, bacterial cell wall constituents such as extracellular lipopolysaccharides and surface appendages act to increase adsorption by reducing fine scale repulsive forces (e.g., van Loosdrecht et al., 1989; Gilbert et al., 1991). The surface physicochemical properties of particle surfaces therefore influences attachment (Mills and Powelson, 1996; Foppen et al., 2010). A principal factor governing interaction with particles is the surface charge of bacteria. Surface charge can influence binding efficacy to sediment surfaces, as chemical interactions in the electrical double layer dominate effective charge and therefore association between sediment and bacteria. *E. coli* and other FIOs typically have an overall net negative surface charge due to the prevalence of carboxyl groups within the cell wall and EPS (Foppen and Schijven, 2006), which could result in attraction or repulsion to strongly positively or negatively charged particles respectively. The surface charge of *Escherichia coli* varies with serotypes suggesting bacteria from different sources could bind differently to sediment (Castro and Tufenkji, 2007; Foppen et al., 2010). Furthermore, Gottenbos et al. (2001) found that bacteria adhered more rapidly to positively charged surfaces but electrostatic interaction impeded bacterial growth after adsorption in pure culture experiments. This interaction decreased the bacterial adenosine triphosphate content and proton motive force upon adhesion (Hong and Brown, 2009) supporting the decreased cell viability identified by van der Mei et al. (2008). Conversely, negatively charged surfaces could promote the opposite, favoring growth of bacteria. Hadjiev et al. (2007) found biofilm attachment is greatest at the maximum surface energy difference between biofilm and material surface. Surface characteristics such as flagellar antigen sites, can vary significantly with species and strain altering the hydrophobicity, electrostatic balance, roughness and surface area parameters of the bacterial surface resulting in markedly different adsorption characteristics to sediment (van Loosdrecht et al., 1987; Stenström, 1989; Bilge et al., 1996; Pachepsky et al., 2009b; Foppen et al., 2010).

Surface characteristics may also be affected by biological aspects such as the metabolic state of the organism. For example, both hydrophobicity and zeta potential (as a measure of wetness) has been shown to be related to the growth rate or phase in *E. coli* (Allison et al., 1990; Smets et al., 1999). A comparison of 17 *E. coli* strains, isolated from livestock or water sources, showed an order of magnitude difference in attachment efficacy when binding to quartz sand, with the most efficient stains concurrently possessing the highest number of genes associated with adhesion, toxin production, iron acquisition, or capsular synthesis (Cook et al., 2011). The mineral chemical and surface composition, organic content and particle size affect the propensity of bacterial cells to adhere or release to the particles (Pachepsky et al., 2009b; Hazen and Sverjensky, 2010). Scholl and Harvey (1992) showed that the mineral surface charge controlled initial adhesion of hydrophilic bacteria. Mineralogy and elemental composition often differs between sediment size fractions, with the smaller particles of the clay fraction providing a larger and more reactive surface area for adsorption (Perkins et al., 2014). Most surfaces are coated in reactive groups such as metals, metal oxides and hydroxides and organic material such as proteins through a process known as surface conditioning (Mills and Powelson, 1996). Quartz tends to have greater adhesion when the isoelectric point (pI) of the compound differs greatly from the point of zero charge of quartz. In contrast, a large difference in pI reduces adhesion in clay minerals (Hazen and Sverjensky, 2010). However, surface properties may not modify the microbial viability post initial adhesion (Busscher et al., 1995). The presence of a conditioning film could mask the impact of surface properties by acting as a barrier to chemical and spatial heterogeneity, for example Lorite et al. (2011) showed that a conditioning film reduces film hydrophilicity and roughness of a material surface, which could influence the rate of subsequent film formation. Alternatively, the film could provide a link between the material surface and bacteria (Singh et al., 2011). The importance of roughness is twofold: firstly, it protects the initial bacteria during adhesion from deleterious effects of shear and second, provides greater surface area for adhesion to occur (van Loosdrecht et al., 1989; Stephenson et al., 2013). Singh et al. (2011) identified a threshold of ~20 nm surface roughness where superior protein adsorption substantially decreased attachment rates and biofilm formation by clogging nanoscale pores on the material surface, although whether this influences adhesion of sediment in the field requires further attention.

Fecal coliforms such as *E. coli* predominantly attach to small particles (< 2 μm), increasing the ease by which they are transported and dispersed in the environment (Muirhead et al., 2006; Goldscheider et al., 2010). Bacterial binding to surfaces, including sediment particles, can be reversible or more permanent (van Loosdrecht et al., 1989; Van Houdt and Michiels, 2005). Fecally derived bacteria are more frequently associated with finer sediments and particles (Chan et al., 1979; Ferguson et al., 1996) than suspended free within the water column (Gerba et al., 1977a; Table 1). Particulate association is important for transport processes: cells attached to larger particles settle to the stream bed, whilst unattached cells, or those attached to small buoyant particles, are likely to be transported further, particularly during storm events (Jamieson, R. C. et al., 2005). Previous research has defined coastal or estuarine sediments as a sink of fecally derived bacteria (Obiri-Danso and Jones, 2000; Deloffre et al., 2005; Berthe et al., 2008; Perkins et al., 2014). Subsequent sediment re-entrainment during storm events, recreational water use, mechanical disturbance and tidal resuspension on mudflats can, therefore, lead to deterioration in microbiological water quality (Crabill et al., 1999).

### Survival of bacterial FIOs in water and sediment

#### Growth and persistence of FIOs and pathogens

The growth of fecally derived bacteria in the environment appears to be restricted mainly to tropical climates or sediments that are subject to intermittent immersion and drying such as riverbank soil, estuaries or coastlines subject to tidal drying and wetting (Table 2) or in the absence of predators (Davies et al., 1995). Maximum decay rates of FIOs in sediments of between −1.1 and −1.3 log_10_CFU/100 g.sediment./d have been reported (Table 2). Although lower inactivation of FIOs of between −0.011 and −0.138 log_10_CFU/100 g.sediment./d and persistence of human pathogens has been reported (Davies et al., 1995). Highly variable survival of *E. coli* in freshwaters has been reported (Table 3) Research suggests sediment associated bacteria exhibit greater survival in marine and river waters compared to free floating bacteria (Roper and Marshall, 1979). For example, the presence of clays resulted in increased *E. coli* survival from phage attack by ~60% (Roper and Marshall, 1974). Particle-bound fecally derived bacteria are partially shielded from most antimicrobial agents or harmful processes such as UV light that might occur in the water. Schultz-Fademrecht et al. (2008), found a 2–4 log increase in FIOs in a streambed biofilm compared to the overlying water column, possibly due to light inactivation in the water but not the sediment or biofilm. In comparative studies, *E. coli* survives longer in sediments containing at least 25% clay (< 2 μm) than in those with larger particles (Burton et al., 1987). Sediment particle size plays a role, with coliforms able to survive for between 76 and 83% longer in sediment comprised mainly of clay particles compared with coarser sediments (Howell et al., 1996). Garzio-Hadzick et al. (2010) showed that fine particulates and organic carbon resulted in slower inactivation in streambed sediments. The composition of clay was also found to impact pathogen survival with goethite reducing viability of pathogenic *E. coli* by 95% compared to other clay types (Cai et al., 2013). The levels of montmorillonite in soil has been associated with reduced occurrence of some human pathogens and greater survival of indigenous bacteria (Filip, 1973); whilst bentonite clays have been shown to inhibit protozoal grazing of *Rhizobium* in liquid culture (Heijnen et al., 1991) and illite clay antagonizes *E. coli* by action of Fe^2+^ ions on the particle surface resulting in loss of outer membrane integrity and therefore viability (Williams et al., 2011; Cai et al., 2013). Future research is necessary to determine whether the elemental/mineral composition of sediment influence the spatial variability of pathogens and fecal indicator bacteria in the environment under representative conditions.

**Table 2:** **Decay or growth of Fecally derived bacteria in coastal and estuarine sediments**.

**Species**	**Habitat**	**Temp (°C)**	**Salinity (PSU)^a^**	**Initial inoculation concentration**	**Decay rates^b^ (log_10_ CFU 100 g or ml d^−1^)**	**References**
*E. coli*	Fecally contaminated estuarine sediment	24	4–6	5 log_10_ MPN 100 ml^−1^	−0.128 (*t* = 4 days) (grew briefly over first 24 h)	Gerba and McLeod, 1976
	Estuarine sediment	24	8–18	~5.5 log_10_ MPN 100 ml^−1^	−0.152 (*t* = 4 days)	
		24	8–18	~9.5 log_10_ MPN 100 ml^−1^	−0.068 (*t* = 17 days)	
	Coastal swash zone beach sediment	17 and 23	24	~6 log_10_ CFU 100 g^−1^	−0.199 (*t* = 9 days)	Korajkic et al., 2013
	Riverbank soil of a tidally influenced tributary	25	10% seawater	~3 log_10_ MPN 100 gDW^−1^	Growth after 12 h exceeded the limits of quantification	Solo-Gabriele et al., 2000
	−0.8% initial moisture content					
	14% initial moisture content			~4 log_10_ MPN 100 gDW^−1^	+0.119 (*t* = 3)	
	34% initial moisture content			<1 log_10_ MPN 100 gDW^−1^	+0.008 (*t* = 3)	
*Fecal colifo*				~4 log_10_ CFU 100 g^−1^	−0.018 (*t* = 28 days)	Davies et al., 1995
				~4.8 log_10_ CFU 100 g^−1^	−0.138 (*t* = 28 days)	
*Fecal streptococcus*				~3.7 log_10_ CFU 100 g^−1^	−0.004 (*t* = 28 days)	
				~4 log_10_ CFU 100 g^−1^	−0.011 (*t* = 28 days)	
Total *Clostridium perfringens*	Marine sediment	22−23	34−35	~4.4 log_10_ CFU 100 g^−1^	+0.001 (*t* = 28 days)	
				~4.8 log_10_ CFU 100 g^−1^	0.000 (*t* = 28 days)	
*C. perfringens* spores				~4.3 log_10_ CFU 100 g^−1^	+0.001 (*t* = 28 days)	
				~4.8 log_10_ CFU 100 g^−1^	0.000 (*t* = 28 days)	
Fecal coliforms*Enterococcus* spp.	Fecally contaminated saltwater sediment	Ambient	~30	5 log_10_ CFU 100 ml^−1^	−1.3 (*t* = 28 days)	Anderson et al., 2005
				5 log_10_ CFU 100 ml^−1^	−1.1 (*t* = 28 days)	
*E. coli* O157	Intertidal sand at coastal beaches	10	34	8.3 log_10_ CFU 100 ml^−1^	−0.136 (*t* = 5 days)	Williams et al., 2007
		10	34	8.3 log_10_ CFU 100 ml^−1^	−0.086 (*t* = 5 days)	
		10	34	8.3 log_10_ CFU 100 ml^−1^	−0.110 (*t* = 5 days)	

PSU, Practical salinity units,

a Salinity units not defined in study (assumed %¸)

b *Bacterial abundance from graphs, normalized per 100 ml or g, and decay rates resolved using the equation from Anderson et al. (2005): r = [ln(N_t_)-ln(N_0_)]/t. Where, N_t_ is the number of bacteria (log_10_ CFU 100 ml or g) at time t; N_0_ is the number of bacteria (log_10_ CFU 100 ml or g) at time 0, and t, time in days. A negative value denotes a decrease in the number of bacteria, whereas a positive value denotes an increase. The decay rates assume exponential decrease and should be applied with caution in sediments (Davies et al., 1995). DW, Dry weight*.

**Table 3:** **Survival of generic and pathogenic ***E. coli*** in water**.

**Species**	**Water source**	**Factor/Variable investigated**	**Measure of decline/survival**		**References**
*E. coli* O157	Sterilized well water (4 sources)	Survival in Different Waters	2 log decline after 35 days		Geldreich et al., 1992
*E. coli* O157 #C4195 and #932	Portable groundwater source	5°C	3.5 log reduction after 70 days		Rice et al., 1992
*E. coli* #R1		20°C	5 log reduction after 35 days		
*E. coli*	Sterile seawater		Rate of die-off under light conditions		Alkan et al., 1995
		Turbidity	Significant effect		
		Sewage	Significant effect		
		Mixing	Significant effect		
		Temperature	Not-significant effect		
*E. coli* K-12 (MC4100)	Filter sterile estuarine water	Organic Matter (Presence/Absence)	Linear regression slopes		Troussellier et al., 1998
		Salinity (Artificial Seawater/Physiological Water) Light (Presence/Absence)	OM+	OM−	
			S – L −+0.050	−0.005	
			S + L −−0.006	−0.020	
			S – L + −0.060	−0.110	
			S + L + −0.120	−0.100	
*E coli* O157	Filtered and autoclaved municipal water, in reservoir water, and in water from two recreational lakes	8°C25°C	1–2 log drop after 91 days Detection limit reached 49–84 days		Wang and Doyle, 1998
*E. coli* O157:H7 (NCTC 12900)	Bottled natural drinking water	Survival in Unsterile mineral water	3 log reduction after 70 days		Kerr et al., 1999
		Sterile mineral water	3.5 log reduction after 70 days		
		Sterile distilled water	4.5 log reduction after 70 days		
*E. coli* O157	River water	Survival in river water	Detection limit reached 27 days		Maule, 2000
*E. coli* O157	Cattle drinking water (2 sources)	Temperature (5 and 15°C) Water source	1 water source—no difference between temperatures		Rice and Johnson, 2000
			2 water source—5°C reached detection limit after 8 days		
			15°C reached detection limit after 4 days		
*E. coli* O157 (Environmental)	Cattle water troughs (473)	Water characteristics that encourage survival	Presence/absence 6/473		LeJeune et al., 2001
*E. coli* O157 #3704 Tn5 *lux CDABE*	Well water from four different sites	Variation in several factors between sites + the presence of different organism	Number of *E. coli* O157 present reduced by copper, predation by protozoa and in competition with other microorganisms		Artz and Killham, 2002
*E. coli* DH5α	Unsterile and sterile groundwater	Influence of microflora	Unsterile T90 2 days		Banning et al., 2002
			Sterile T90 82 days		
*E. coli* O157:H7 (NCTC 12900)	River water—with and without feces Sterile distilled water	Difference between temperature and water sources	River water w/o feces—outside <15°C—detection Limit reached after 14 days		McGee et al., 2002
			− inside 15°C—Detection limit reached after >31 days		
			River water w/o Faeces—Outside <15°C—Detection limit reached after 24 days		
			Outside <15°C—Detection limit reached after 17 days		
			Inside 15°C—2.5 log drop after 31 days		
*Escherichia coli* Famp (ATCC 700891)	Dechlorinated water	10°C	*D*-Value 7.7 Days		Allwood et al., 2003
		22°C	*D*-Value 5.7 Days		
		37°C	*D*-Value 3.0 Days		
*E. coli* O157 #3704 Tn5 *lux CDABE* and *E. coli* O157 #3704	Sterile artificial groundwater	Difference between the strains at 15°C	Both showed a 5-log drop over 70 days		Ritchie et al., 2003
*E. coli* O157	Surface water from lakes and rivers	6°C	Detection limit reached 32–51 Days		Czajkowska et al., 2005
		24°C	Detection limit reached 21–32 Days		
7 strains of *E. coli* O157	Untreated well water	10°C	2 strains—1–2 log drop after 56 days		Watterworth et al., 2006
			2 strains—4 log drop after 56 days		
			3 strains—detection limit reached <42 days		
		22°C	1 strain—6–7 log drop after 56 days		
			2 strains—detection limit reached <56 days		
			4 strains—detection limit reached <42 Days		
*E. coli* O157 #3704	Non-sterile: lake Fecally contaminated puddle River Drinking trough	Variation in several factors between sites	T_99_ 12.9 days		Avery et al., 2008
			T_99_ 17.8 days		
			T_99_ 6.0 days		
			T_99_ 6.3 days		
*E. coli* O157:H7	Pond and holding tank water	Difference between water sources	Pond—detection limit reached after 33 days		Suhalim et al., 2008
			Holding tank—detection limit reached after 69 days		
6 clinically isolated ETEC strains	Sterile-filtered sea water and freshwater	Induction of VBNC state in water	2 log drop after 12 weeks		Lothigius et al., 2010

#### Biofilm formation

**Figure 1:**
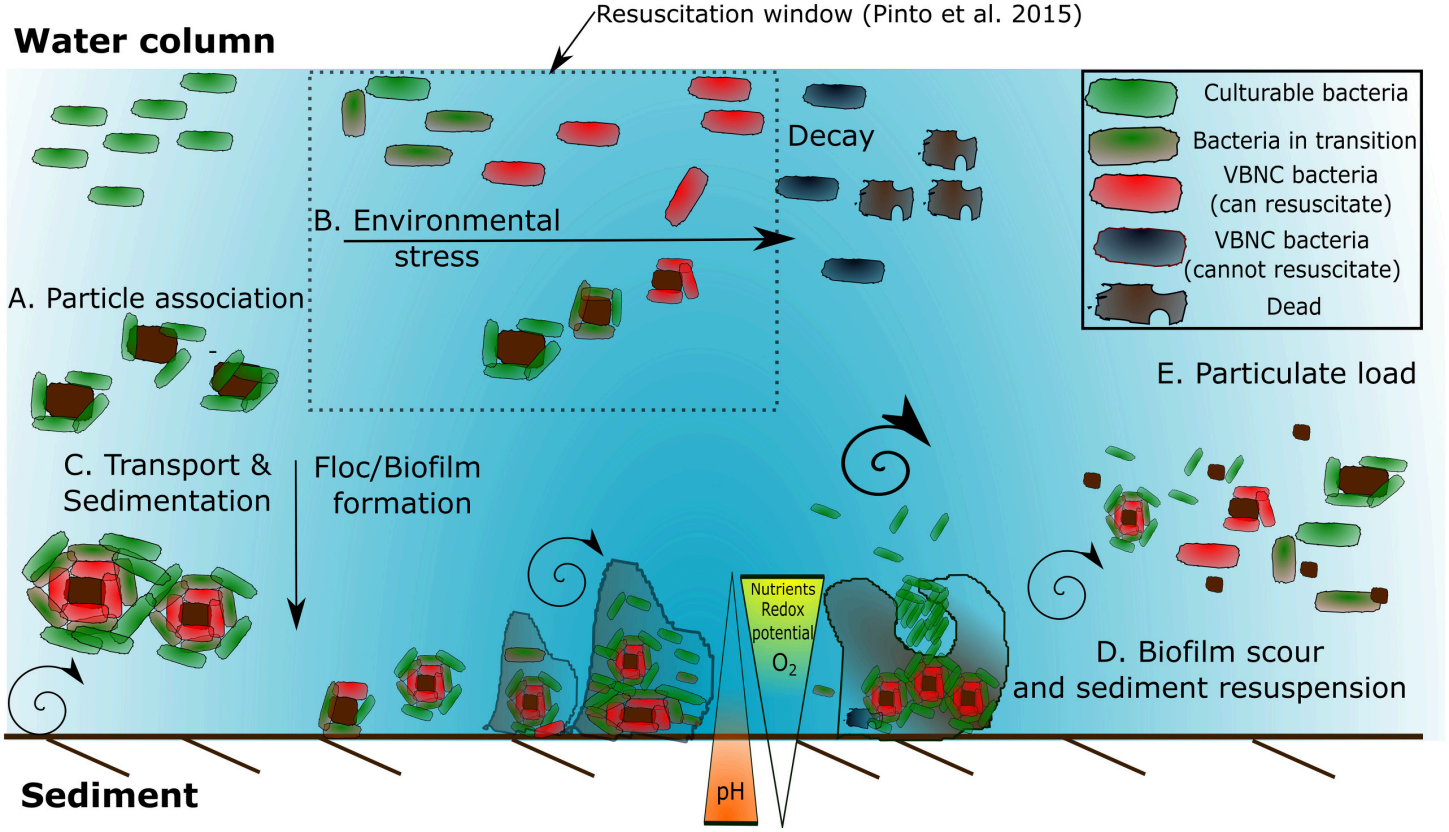
**Factors stimulating bacterial accumulation in the environment, induction to and resuscitation from VBNC state. (A)** Bacterial-particle association and bacteria-bacteria association. **(B)** Environmental stressors such as high/low nutrients, oxygen, redox potential, and oxidative stress induce biofilm formation. **(C)** Transport and sedimentation provides a downward flux to sediment. As the biofilm grows on the sediment the mass transfer rate is no longer sufficient resulting in localized gradients in electron acceptors and nutrients. This results the induction of VBNC bacteria. **(D)** High flow events result in shear and can slough the biofilm, reducing the stabilizing effect of the EPS. **(E)**. This can further exacerbate the resuspension of bacteria within the water column leading to increased particulate load (Adapted from Ayrapetyan et al., 2014b; Pinto et al., 2015).

Biofilm formation is an important microbial survival strategy in aquatic systems and biofilms are produced when nutrients are abundant (Costerton et al., 1995). Typically, biofilm formation consists of five stages (Van Houdt and Michiels, 2005). The first stage is a reversible association/attachment between the bacterium and the solid surface when brought together by flow of the medium (Figure 1A). This particle association can improve bacterial survival under stressful conditions (Figure 1B). The second stage of biofilm formation is the production of EPS, an important bacterial surface determinant of attachment and fimbriae that anchor the bacterium irreversibly to the solid surface (Junkins and Doyle, 1992; Figure 1C). During the third and fourth stages, the structure of the EPS matrix matures with the addition of macromolecules such as proteins and deoxyribonucleic acid (DNA) (Sutherland, 2001). The fourth stage is distinguished by the alteration of the biofilm to trap and funnel nutrients to those bacteria immobilized in that matrix. The final stage is the steady release of bacteria from the fully mature biofilm, which can occur through shear or sediment resuspension (Figure 1D). It is thought that quorum sensing plays a determinate role in biofilm formation (Costerton et al., 1995) and the response of bacteria to high velocity fluid flow which varies at the transcriptional level (Kim et al., 2016). Further work could elucidate the role of quorum sensing and FIO abundance in sediments.

Intertidal mudflats are comprised of very small silt and clay particles deposited when low energy currents and wave action prevails (Stal and de Brouwer, 2003). The stabilization of the sediment is due to a combination of compaction of the sediment during periods of drying (Stal and de Brouwer, 2003) and through the release of EPS by diatoms and bacteria creating stabilizing biofilms (Madsen et al., 1993). Enteric organisms such as *E. coli* (pathogenic strains), *Campylobacter* spp., *Salmonella* spp. and the pathogenic protozoan *Cryptosporidium parvum* are known for both creating and colonizing existing biofilms in drinking water systems (Wingender and Flemming, 2011). *Enterococcus* spp. form biofilms in beach sand (Piggot et al., 2012), whilst non-pathogenic *E. coli* are known to persist or even grow within coastal and estuarine environments, particularly in tropical/subtropical climates (Byappanahalli and Fujioka, 1998) and non-pathogenic *Clostridium* spp. have been isolated from an estuarine mudflat (Villanueva et al., 2007). At locations where significant fecal contamination occurs, FIOs/pathogens can colonize existing biofilm communities. Enteropathogenic *E. coli* O157:H7 is known to produce biofilms on a range of solid surfaces such as plastic, steel, wood, plant roots and leaves, facilitating long-term survival in the environment (Cooper et al., 2007). The ability of *E. coli* O157:H7 to produce biofilms, however, was dependent on the presence of other bacteria (Bauman et al., 2009; Klayman et al., 2009), and it is likely that surface roughness and the age of the biofilm are major determinants for survival (Korber et al., 1997). Biofilms have also been shown to be a reservoir for enteric viruses, suggesting that these entities persist longer in biofilms than in drinking water and wastewater (Skraber et al., 2005, 2009). Biofilms have been shown to provide protection from the surrounding environment, such as from antimicrobial compounds (e.g., chlorine) and UV exposure (Quignon et al., 1997; Ryu and Beuchat, 2005), and can enhance the infectivity of some organisms such as *Legionella* spp. (Wingender and Flemming, 2011); thus, facilitating persistence of these organisms and viruses. Another important aspect of biofilms is their potential for harboring bacteria in the VBNC state (Bryers, 2000; Schultz-Fademrecht et al., 2008; Wingender and Flemming, 2011). Therefore, quantifying the survival of bacteria in the environment is not a straightforward exercise.

#### Metabolic activity of fecal bacteria

Fecally derived bacteria are introduced into the aquatic environment through surface run off, wastewater discharge or direct defecation. However, the viability, persistence and metabolic activity within or between indicator species is not constant in the environment (Anderson et al., 2005). For example, the metabolic activity of a bioluminescent strain of *E. coli* O157:H7 decreased due to exposure to salt water, whilst elevated nutrients boosted its microbial activity (Williams et al., 2007) possibly resulting in growth (Shelton et al., 2014) or reduction in inactivation (Garzio-Hadzick et al., 2010). In most fresh and marine waters, metabolic activity rapidly declines after release from feces, which may result from insufficient carbon source or absence of host factors (Thorn et al., 2011; Li et al., 2014). Knowledge of the physiological state of *E. coli* is particularly important, as inactive cells (stationary phase), possess greater resistance to environmental stresses such as acidity and anoxia, thereby increasing the probability of survival (Cheville et al., 1996; Saby et al., 1999). Experimental evidence shows that in the log phase, *E. coli* O157:H7 was more vulnerable to biocides and environmental stress (Arnold and Kaspar, 1995); however, if the environment is suitable for growth, this facilitates rapid resource exploitation and proliferation. Current evidence suggests that *E. coli* O157:H7 enters a stationary phase after detachment from intestinal margins in ruminants (Poulsen et al., 1995). Subsequently, the bacterium leaves its host in the stationary phase, increasing its chances of survival in the environment. *E. coli* from cattle feces was shown to be in the VBNC state prior to any environmental exposure (Wu et al., 2009b), suggesting a large fraction of the fecal indicator population may be recalcitrant but non-culturable in agricultural sources when enumerated by conventional microbiological plate counting.

#### Viable but non-culturable (VBNC) state of fecal indicators and pathogens

VBNC bacteria are defined as cells that are in a state of low metabolic activity, and are therefore viable, but are unable to be cultivated on solid selective microbiological culture media; however, under favorable conditions, VBNC cells may resuscitate and regain the ability to grow on microbiological media. The VBNC state is therefore an important methodological limitation, thus preventing the representative enumeration of bacterial abundance in the environment and clinical settings by microbiological plate count analysis (Oliver, 2010). Under sub-optimal conditions such as starvation, salinity, electron acceptor conditions, temperature or pH bacteria enter a “dormant” state. Return of optimal conditions may result in resuscitation (Oliver, 2005). Therefore, standard water quality monitoring surveys do not adequately represent this sub-population of fecally associated VBNC bacteria/pathogens within the water. Recently, studies have examined VBNC FIOs in sediments and biofilms. These environments tend to be deficient in a growth limiting electron acceptor or nutrient and therefore facilitate a greater proportion of VBNC bacteria than expected in free floating systems (Bryers, 2000; Amel et al., 2008; Lieleg and Ribbeck, 2011). For example, greater numbers of *E. coli* and *Salmonella* have been isolated from sediments by molecular methods, than recorded by culturing techniques, indicating that these bacteria could enter the VBNC state in sediments (Amel et al., 2008; Berthe et al., 2008; Luna et al., 2010). In addition, dissolved nucleic acids are more readily extracted than particulate forms which could represent a bias for enumeration (Paul et al., 1991). *Vibrio* spp. are frequently used as model organisms for VBNC studies and enter and recover from the VBNC state under a variety of different stimuli (see: Oliver and Bockian, 1995; Oliver et al., 1995; Amel et al., 2008; Li et al., 2014; Pinto et al., 2015 for different stimuli). In contrast, studies on sediments are sparse, for example, Amel et al. (2008) found that *V. fluvialis* entered the VBNC state in sediments and could be resuscitated even after 1 year. Fukushima and Seki (2004) and Randa et al. (2004) challenge the VBNC notion by suggesting that extremely low abundance of suspended *V. vulnificus* and *V. parahaemolyticus* in winter months is due to the sediment acting as a microbial reservoir, as opposed to the bacteria entering VNBC. Further, Fukushima and Seki (2004) highlight that the proliferation of *Vibrio* spp. after a water temperature increase is due to the replication and release of the daughter cells in the sediment or biofilm rather than the resuscitation of cells from the VBNC state in the water column. Lee et al. (2007) found that drinking water pipe material composition was critical in governing the relative proportion of VBNC and culturable bacteria. However, further research is required on methods to enumerate the numbers of fecally associated bacteria entering the VBNC state in sediments (Amel et al., 2008). Delineating resuscitation from growth remains a significant challenge for the use of direct approaches (Ayrapetyan et al., 2014a; Ramamurthy et al., 2014, Table 4). Physiochemical factors governing induction to and resuscitation from VBNC in biofilms requires further attention, particularly on methodologies to sample VBNC bacteria in sediments/biofilms non-destructively.

**Table 4:** **Comparison of methods to enumerate viable but non-culturable (VBNC) bacteria–suitability for sediment**.

**Technique**	**Direct or indirect**	**Method aim and calculation required**	**Advantages**	**Disadvantages**	**Suitable for sediment?**	**References**
Nalidixic acid	Indirect	Nalidixic acid is bacteriostatic and inhibits cell division at low concentrations.	Allows differentiation of dividing viable cells from non-dividing VBNC cells.	“Resuscitated” cells would subsequently be inhibited by Nalidixic acid as they start to grow.	No	Ohtomo and Saito, 2001
		Need to run in conjunction with direct counts to ensure total counts do not change.		Some bacteria are Nalidixic acid-resistant.		
		**VBNC = (live-un-elongated cells under Nalidixic acid treatment)**		“Injured” cells may only grow on non-selective media, difficult on sediment samples.		
*Bac*Light™	Indirect	Uses two dyes (SYTO9 and Propidium iodide) to stain live cells green and dead cells red. Cells are counted under the microscope.	Membrane integrity is one of the most conservative estimators of viability. Can result in overestimation of viability and VBNC fraction.	Step for disaggregation from sediment required.	No	Hassard et al., 2014
				Assumes that dead cells have disrupted membranes.		
				Cells may form clusters and be difficult to count.		
		**VBNC = (live – culturable)**				
Fluorescent in-situ hybridisation (FISH) and peptide nucleic acid FISH (PNA-FISH).	Indirect	Oligonucleotide probes hybridise to target DNA/RNA and fluoresce under the microscope.	Relatively straight-forward technique. PNA probes have a superior binding capability than traditional FISH probes.	Need to find a species/strain-specific nucleotide probe.	No	Halpern et al., 2007; Malic et al., 2009
				Reliant on microscope quantification		
		**VBNC = (FISH positive – culturable)**.		Cells may form clusters and be difficult to count.		
Immunomagnetic separation	Indirect	Antibodies for a specific species or strain are coated onto magnetic beads.	Not a standalone method for VBNC detection, Immunomagnetic separation can be used to isolate the organism of choice from environmental samples in conjunction with a quantification method.	Not 100% specific	Yes	Gwyther et al., 2013
		A magnet is used to pull the bacteria-linked beads from an environmental sample.		Step for disaggregation from sediment is required.		
				Adds an extra step into the analysis time.		
			Relatively straight-forward technique.	Quantification method required.		
Flow cytometry (FCM)	Indirect	Cells are labeled with nucleic acid stains e.g., *Bac*Light™. The flow cytometer sorts each cell individually, based on fluorescence backscatter which is used to determine abundance of live and dead cells.	FCM can distinguish between reproductively viable, metabolically active, intact and permeabilized cells.	Requires pure cultures or the quantification of entire populations.	Yes	Wallner et al., 1995; Khan et al., 2010
				Assumes that dead cells have disrupted membranes.		
				Rapid *in-situ* analysis of single cells.		
		**VBNC = (live – culturable)**		Difficult to distinguish between bacteria and phages if using environmental samples due to overlap of distributions and signal noise.		
				FCM-FISH has been applied with limited success.		
Propidium monoazide—quantitative PCR (PMA-qPCR)	Indirect	PMA binds to DNA in membrane-compromised cells, preventing DNA replication during PCR.	Can target specific species/strain of bacteria.	PMA-qPCR does not in itself distinguish VBNC cells, but enumerates the number gene equivalents from the bacteria with intact membranes.	Yes	Nocker et al., 2007; Gin and Goh, 2013
		ΔCTrefers to the difference in qPCR threshold cycles CT between total bacteria and live bacteria	qPCR is quantitative.	Detachment from sediments and particulate matter required as a pretreatment.		
		**VBNC = (PMA negative gene equivalents – culturable)**.				
Ethidium monoazide—loop mediated isothermal amplification (EMA-LAMP).	Indirect	EMA binds to DNA in membrane-compromised cells, preventing DNA replication.	Can target specific species/strain of bacteria.	Similar limitations as PMA-qPCR	Yes	Wang et al., 2012,
			LAMP is quicker than PCR.			
		**VBNC = (EMA negative gene equivalents – culturable)**.				
Reverse transcription-quantitative polymerase chain reaction (RT-qPCR).	Indirect	Quantitative PCR method used to detect expression levels of RNA e.g. *rpoS* gene mRNA.	Targets RNA expression, which is a proxy for activity in bacteria.	Environmental matrices, particularly sediment, may contain PCR inhibitors which restrict applicability.	Yes	Yaron and Matthews, 2002; Quilliam et al., 2011b; Wingender and Flemming, 2011
			Can detect target genes active in VBNC bacteria and compare to levels in culturable bacteria referenced against housekeeping genes.	Difficult to extract RNA. Requires suitable sampling regime and storage.		
Autoinducers (AI) /resuscitation promotion factors (RPF).	Direct	Synthetic or biologically produced autoinducer 2 (AI2) or RPF to measure culturability of exposed and unexposed population.	Quantifies VBNC bacteria using the same quantification methodology as ‘culturable’ bacteria therefore a representative comparison.	Difficult to distinguish VBNC from additional growth due to autoinducer/RPF.	Yes	Bari et al., 2013; Ayrapetyan et al., 2014a
				Additional nutrients/cofactors may be required.		
		**VBNC = (AI or RPF culturable)- (normal culturable)**.	Species specific or broad spectrum RPF can be used depending on requirements.			
				Often species specific.		
				Theoretical distinction between resuscitation and growth based on growth rates.		
Pre-rRNA analysis molecular viability testing.	Indirect	Detects innate synthetic activity of rRNA precursors.	Greater fraction of RNA pool than mRNA so easier to detect.	Possibility for false negatives lack of detection = VBNC cells.	Yes	Cangelosi et al., 2010,
				Requires a measure of abundance of species of interest.		
		Direct indicator of growth as pre-rRNAs only formed in growing cells. In dormant cells pre-rRNA levels decline.	Species specific or constitutive precursors can be selected based on required resolution.			
		After nutrient stimulation there is a ratiometric increase in abundance of pre-rRNA from viable cells but not non-viable cells.	Can be used to separate resuscitation from growth, as response time is quicker than that of growth rate of bacteria.	Requires nutrients to stimulate response.		
				Reliant on RT-qPCR for detection so similar limitations at quantification step.		
Dilution to extinction—resuscitation potential	Direct	VBNC bacteria can be distinguished from growth by a serial dilution method. Cells are subjected to a log dilution series below 1 CFU/ml of culturable cells. These diluted cells are cultured, if growth is determined then bacteria have resuscitated from VBNC.	Quantitative, utilises the same methodology to determine VBNC as culturable counts therefore directly comparable.	Dilution could inhibit quorum sensing based resuscitation.	Yes	Zhang et al., 2015
		**VBNC = Resuscitated counts – culturable counts**				

*Bold indicates the factors used to calculate VBNC*.

Indirect approaches such as microscopy combined with live/dead staining, taxon-specific fluorescent *in situ* hybridization (FISH) and qPCR have all been utilized for measurement of VBNC bacteria in environmental samples by comparing “total” or “live” bacteria with “culturable counts” (Table 4). Indirect methods for VBNC quantification bacteria in environmental water samples are also not appropriate for sediments due to the 3D nature of the matrix, extracellular polymers and blocking of incident light for methods such as *Bac*Light™ staining. Direct methods (utilizing microbiological plate counts) such as the application of resuscitation promotion factors (e.g., autoinducers) have been shown experimentally to be useful for measuring the total bacterial population including the VBNC fraction in water but have yet to be applied to sediment (Atkinson and Williams, 2009; Bari et al., 2013; Ayrapetyan et al., 2014a). The principal issue for these approaches is delineating resuscitation of existing bacteria from growth of daughter bacteria (Ayrapetyan et al., 2014b) and this problem remains with sediment. The phenotypic changes that occur in the VNBC state can be assessed using reverse transcription quantitative PCR (RT-qPCR; Table 4) as alterations to membrane lipid composition, fluidity and a rearrangement of the outer membrane composition have been reported previously (Scherber et al., 2009). Membrane changes in response to stress are modulated via the osmosensor protein EnvZ, which is sensitive to changes in external solute concentration. This cascade is potentially regulated by MzrA, and upregulation increases outer membrane proteins such as *ompW* (Asakura et al., 2008; Darcan et al., 2009). The porin protein encoded by *ompW* gene is known to be upregulated by extremes of pH (Wu et al., 2009a), whilst *E. coli* osmoregulation proteins OmpC/F production are regulated by changes to solute concentration. This is an important survival strategy for coastal and transitional zones, such as estuaries (Rozen and Belkin, 2001). The analysis of pre-ribosomal RNA (pre-rRNA) has received interest recently (Cangelosi et al., 2010). Reported advantages include greater relative abundance of pre-rRNA compared to messenger RNA (mRNA) so response is quicker, which subsequently allows separation of resuscitation from growth (Table 4). The method relies on the ratiometric increase in pre-rRNA levels in bacteria subject to a nutrient-based resuscitation compared to a control in the absence of nutrients; this provides a dormant to non-dormant ratio (Cangelosi et al., 2010). It is still unclear if this approach is valid for sediments.

Viable but non-culturable *Pseudomonas* spp. exhibited a reduction in nutrient transport, respiration rates and macromolecular synthesis compared to culturable equivalents; however these VBNC cells can still actively divide at a reduced rate (Peneau et al., 2007). Adhesion to the external surface of zooplankton also stimulates fecal enterococci to enter a VBNC state (Signoretto et al., 2004) and this may form a vital part of the transmission pathway (Cellini et al., 2005). Favorable growth conditions and an ideal stoichiometric ratio of carbon to inorganic elements enables recovery from VBNC state, although the resuscitation rate is highly variable depending on species and conditions studied (Arana et al., 2007; Bari et al., 2013; Ayrapetyan et al., 2014a) and may take days to occur (Scherber et al., 2009). Reversion to a culturable state probably involves a resuscitation-promoting or anti-dormancy factor which can cleave peptidoglycan, altering the mechanical properties of the cell wall to facilitate cell division or release lysis products that function as anti-dormancy signals (Ward et al., 2006). Whether VBNC cells are capable of causing infection is poorly understood, and is dependent on the reactivation time, external conditions and if additional vectors/cofactors are required or involved prior to infection. Research into *Salmonella* has indicated that newly formed VBNC cells do not mount a strong infection response (Passerat et al., 2009) possibly due to lack of suitable resuscitation factors. The resuscitation window is defined as the time or amount of stress a VBNC bacteria can undergo and still resuscitate. If conditions remain unfavorable, then VBNC bacteria go beyond the period where resuscitation can occur, and are considered injured, but may still be viable. Finally, eventual death may occur (Pinto et al., 2015; Figure 1). Zhang et al. (2015) utilized a method known as dilution to extinction (Table 4) and showed that *E. coli* had significant resuscitation potential after UV treatment, suggesting that routine disinfection induces the VBNC state as opposed to cell death in bacteria. Whether bacterial FIOs and pathogens have “resuscitation potential” which could represent a risk to public health or water quality requires further attention.

The potential for bacteria to enter the VBNC state suggests that sediments may be a greater store of fecally-derived bacteria than previously quantified. Sediments and biofilms provide distinct gradients of nutrients, electron acceptors and pH, whilst protecting from some environmental stressors, such as shear and light (Bryers, 2000). Additional methodological improvements are required to reliably quantify VBNC bacteria in sediment. Gene targets which are expressed and specific to the VBNC response can be used in combination with RT-qPCR quantification, providing a useful approach for VBNC analysis in sediment. This is because probes may be species/strain-specific and are based on the production of messenger RNA (mRNA) or pre-rRNA molecules which are short-lived and can provide high resolution information on temporal gene expression (Yaron and Matthews, 2002; Cangelosi et al., 2010). However, the low extraction efficiency of RNA and downstream qPCR inhibition which is a particular challenge in sediment needs to be overcome (Miura et al., 2011; Carreira et al., 2015).

## Fate and behavior of fecally derived viruses in sediments

There has been considerable attention attributed to the fate and transport of viruses in environmental matrixes, such as soil, groundwater and surface water (Schijven and Hassanizadeh, 2000; John and Rose, 2005; Sen and Khilar, 2006). The main factors affecting viral adsorption and persistence in porous media include the type of virus and media, temperature, pH, ionic strength and the presence of organic matter (Jin and Flury, 2002). The dominant mechanisms are well-understood in porous media, however, little information is available on their importance in sediment. Enteric viruses readily adsorb to many types of sediment with reported adsorption rates of between 37 and 100% (Carlson et al., 1968; Gerba et al., 1977b; LaBelle and Gerba, 1979; Gerba et al., 1980; Bitton et al., 1982; Tsai et al., 1983; Johnson et al., 1984). The high adsorption levels in estuarine and marine sediment (Table 5) may be attributed to the high organic content and hydrophobicity of the sediment particles (Chrysikopoulos and Syngouna, 2012). Other factors shown to influence viral adsorption to porous media may have limited impact in sediment due to the production of conditioning films. However, the physico-chemical properties of viral particles and water may play a role in viral adsorption-desorption kinetics in sediment. For instance, Bitton et al. (1982) found complete adsorption (100%) of poliovirus to marine sediment compared to lower adsorption to freshwater sediment (37–45%). LaBelle and Gerba (1979) showed that increased salinity and decreased pH enhance the desorption (5–10%) of echovirus from estuarine sediment, whereas the desorption of other enteric viruses (rotavirus, poliovirus, and coxsachieviruses) was not affected by those changes. Carlson et al. (1968) found that the presence of bivalent cations in solution enhanced viral adsorption to clay, whereas albumin promoted desorption. These results imply that enteric viruses may desorb from sediment when conditions change, for example to heavy rainfalls or tidal changes.

**Table 5:** **Fecally-derived virus adsorption to sediment**.

**Virus type**	**Sediment type**	**Adsorption (%)**	**References**
Poliovirus 1	Marine (99.7% sand, 0.3% clay)	99%	Bitton et al., 1982
	Marine, organic muck	100%	
	Estuarine (20.7% sand, 24.88% clay, 54.4% silt, 3.8% organic matter)	~100	LaBelle and Gerba, 1980
	Estuarine	99.9	Gerba et al., 1980
	Estuarine (mud and sand)	99.2–99.98	Gerba et al., 1977b
	Estuarine (99% sand, 1% silt)	93.4	Johnson et al., 1984
	Estuarine (52.3% sand, 30.3% silt, 17.4% clay)	99.8	
	Estuarine (89.3% sand, 6% silt, 4.6% clay)	98.3	
	Estuarine (37.3% sand, 39.2% silt, 23.5% clay)	99.9	
	Estuarine (10.1% sand, 48.2% silt, 41.7% clay)	>95	Tsai et al., 1983
	Estuarine (79.2% sand, 11.8% silt, 9.1% clay)	>95	
	Freshwater (99.6% sand, 0.4% clay)	37%	Bitton et al., 1982
	Freshwater (99.7% sand, 0.3% clay)	45%	
Coxsackievirus B1	Estuarine (99% sand, 1% silt	64.6	Johnson et al., 1984
	Estuarine (52.3% sand, 30.3% silt, 17.4% clay)	98.4	
	Estuarine (89.3% sand, 6% silt, 4.6% clay)	98.6	
	Estuarine (37.3% sand, 39.2% silt, 23.5% clay)	99.0	
Coxsackievirus B3	Estuarine (20.7% sand, 24.88% clay, 54.4% silt, 3.8% organic matter)	100	LaBelle and Gerba, 1980
	Estuarine	99.8	Gerba et al., 1980
	Estuarine (10.1% sand, 48.2% silt, 41.7% clay)	>95	Tsai et al., 1983
	Estuarine (79.2% sand, 11.8% silt, 9.1% clay)	>95	
Coxsackievirus B4	Estuarine	95	Gerba et al., 1980
Echovirus 1	Estuarine (20.7% sand, 24.88% clay, 54.4% silt, 3.8% organic matter)	90	LaBelle and Gerba, 1980
	Estuarine	87.0–99.99	Gerba et al., 1980
Echovirus 7	Estuarine	>99.99	
Echovirus 29	Estuarine	>99.99	
Echovirus 11	Estuarine (99% sand, 1% silt	66.6	Johnson et al., 1984
	Estuarine (52.3% sand, 30.3% silt, 17.4% clay)	98.9	
	Estuarine (89.3% sand, 6% silt, 4.6% clay)	99.0	
	Estuarine (37.3% sand, 39.2% silt, 23.5% clay)	99.5	

Gerba et al. (1980) observed species/strain specific differences in viral adsorption to sediment, suggesting that capsid properties may play an important role in adhesion. The physico-chemical characteristics of viral particles, e.g., pI, hydrophobicity and capsid structures have been shown to play an important role in the adsorption of viruses to porous media. Dowd et al. (1998) highlighted the influence of viral pI on the adsorption rate of viruses, where a smaller pI (3.9–5.3) showed more adsorption than a larger pI (6.6–7.7) despite examined viruses being of similar sizes. Farkas et al. (2015) observed that the adsorption of rotavirus viral surrogates with similar size and pI adsorbed differently to hydrophobic media. Further differences were found in the adsorption of viral surrogates with similar size, zeta potential and hydrophobicity to porous media, suggesting that the composition of viral capsid also affects viral adhesion (Pang et al., 2014; Farkas et al., 2015). Further, Samandoulgou et al. (2015) found that extremes of pH and temperature can change the mechanism of norovirus association with sediment from electrostatic to predominantly hydrophobic, as loss of ordered molecular structure in the protein head results in an increase in hydrophobic attachment sites resulting in greater adsorption of norovirus. Hydrophobic interactions of proteins are enhanced by high salinity thus viral attachment/detachment kinetics in estuarine environments may change rapidly.

From a public health perspective, the inactivation of enteric viruses in sediment is also important. However, most studies focus on the presence/absence and concentration of enteric viruses in sediment and little is known about the inactivation and degradation of viral particles. Viruses in the water column are inactivated at a faster rate than in sediments (Smith et al., 1978; LaBelle and Gerba, 1980; Liew and Gerba, 1980; Rao et al., 1986b), indicating that sediments confer protection for viruses from degradation. The persistence of viruses is largely dependent on sediment and virus type. For instance, coxsachievirus degradation ranged from 0.2 to 2.5 log in three types of sediment in 20 days, whereas poliovirus and echovirus degraded by 0.5–4 log and 2–4 log, respectively (Table 6). As in water, microbial activity enhances the degradation of enteric viruses in the sediment, whereas small changes in temperature and salinity have little effect on inactivation. Inactivating substances, such as enzymes, may also adsorb to particles and thus have no effect on viral degradation (Gerba and Schaiberger, 1975). Interestingly, virus inactivation increased in polluted water even in the absence of microorganisms (LaBelle and Gerba, 1980) probably due to reaction with humics in water. Viral adsorption to sediment particles has also been shown to increase viral thermostability, possibly explaining the recalcitrant nature of enteric viruses in sediments (Liew and Gerba, 1980).

**Table 6:** **Persistence of Fecally-derived viruses in coastal and estuarine sediments**.

**Viral strain**	**Habitat**	**Temp (°C)**	**Salinity (PSU)^a^**	**Initial inoculation**	**Reduction**	**References**
Coxsackievirus B3 Nancy	Estuarine sediment (mud, shell) and seawater			~7 log10 PFU 100 ml^−1^	~3.8 log in 20 days	Smith et al., 1978
	Estuarine sediment (sand) and seawater			~7 log10 PFU 100 ml^−1^	~4 log in 20 days	
	Estuarine sediment (mud, sand) and seawater			~7 log10 PFU 100 ml^−1^	~2.7 log in 20 days	
Echovirus 1 Ferouk	Estuarine sediment (sand) and seawater	30	25	~7 log10 PFU 100 ml^−1^	~4 log in 10 days	
	Estuarine sediment (mud, shell) and seawater			~7 log10 PFU 100 ml^−1^	~4 log in 18 days	
	Estuarine sediment and seawater			7-8 log10 PFU 100 ml^−1^	~1 log in 6 days	LaBelle and Gerba, 1980
	Estuarine sediment (mud, sand) and seawater			~7 log10 PFU 100 ml^−1^	~1.5 log in 20 days	Smith et al., 1978
Poliovirus 1 LSc	Estuarine sediment (mud, shell) and seawater			~7 log10 PFU 100 ml^−1^	~4 log in 14 days	
	Estuarine sediment (mud, sand) and seawater	31		~7 log10 PFU 100 ml^−1^	~4 log in 18 days	
	Estuarine sediment (sand) and seawater		25	~7 log10 PFU 100 ml^−1^	~4 log in 7 days	
	Estuarine sediment and seawater	31	30	7–8 log10 PFU 100 ml^−1^	~2.5 log in 7 days	LaBelle and Gerba, 1980
		33	26	7–8 log10 PFU 100 ml^−1^	~ 3.3 log in 6 days	
		33	27	7–8 log10 PFU 100 ml^−1^	~2.8 log in 6 days	
	Estuarine sediment and artificial seawater	4		~6 log10 PFU 100 ml^−1^	Stable for 33 days^b^	Liew and Gerba, 1980
		24		~6 log10 PFU 100 ml^−1^	~3 log10 over 33 days	
		37		~6 log10 PFU 100 ml^−1^	~2 log in 4 days	
	Estuarine sediment and seawater	20–25	2–20	7.8 log10 PFU 100 ml^−1^	7.3 log in 19 days	Rao et al., 1984
	Estuarine suspended solids and seawater	20–25	2–20	7.5 log10 PFU 100 ml^−1^	5.8 log in 19 days	
	Estuarine “fluffy” sediments and seawater	20–25	2–20	7.6 log10 PFU 100 ml^−1^	6.5 log in 19 days	
Simian Rotavirus SA11	Estuarine sediment and seawater	20–25	2–20	7.7 log10 PFU 100 ml^−1^	4.7 log in 19 days	
	Estuarine suspended solids and seawater	20–25	2–20	7.9 log10 PFU 100 ml^−1^	3.9 log in 19 days	
	Estuarine “fluffy” sediments and seawater	20–25	2–20	7.6 log10 PFU 100 ml^−1^	5.6 log in 19 days	

a *Salinity assumed PSU = %0*.

*~Prefix represents viral abundance estimated from graphs*.

Viruses may reversibly attach and detach from sediment and re-enter the water column or the sediment-associated viral particles may be transported from polluted to non-polluted waters. Hence, viruses entering the water body from sediment may increase the public health risk. Furthermore, due to water turbulence the viral particles attached to less dense sediment may be easily resuspended. Rao et al. (1986b) implied that solid-associated rotavirus may be transported >5 km (≥3 miles) in estuarine water. Furthermore, sediment-associated viruses may be taken up by shellfish or crustacea that are destined for human consumption. Despite the risks of viral presence in sediment being well-recognized, little is known about the fate of viruses in sediment, and especially the factors which may affect viral adsorption and inactivation *in situ* e.g., sunlight, chemical contamination and organic matter. This is pertinent when considering the impact of sediment/particle association on the suitability of using viruses as regulatory indicators for drinking waters and bathing/shellfish water quality (Bosch et al., 2008).

### Methods for the enumeration of fecally derived viruses from sediments

The identification and quantification of enteric viruses in the environment is challenging mainly due to the lack of reliable methods for accurate quantification and the difficulty in eluting viruses from sediment. The most frequently used methods for quantification of enteric viruses in environmental studies are tissue culture, electron microscopy (EM), enzyme-linked immunosorbent assay (ELISA), flow cytometry and qPCR or RT-qPCR (Weinbauer, 2004; Duhamel and Jacquet, 2006).

Traditional tissue culture approaches involve incubation of virus-containing samples with suitable host cell lines that allow viral replication. The cytopathic effects (host cell damage) can be observed under the light microscope (Dulbecco, 1952; Moce-Llivina et al., 2004). For viruses which do not lyse host cells, a focus-forming assay is used which involves the use of fluorescent antibodies that bind to viral antigens allowing the detection of clusters of infected cells (foci) by fluorescent microscopy (Payne et al., 2006). Nonetheless, culture-based assays can take weeks to perform (Storch, 2000) and often underestimate the number of viruses due to viral aggregation; however, as loss of infectivity is permanent, this provides a useful estimate of infectivity decay rates (Charles et al., 2009). Furthermore, some enteric viruses such as human noroviruses and sapoviruses cannot be maintained *in vitro*, hence they cannot be quantified by culture. Intact virus particles after incubation with an appropriate dye can be visualized using EM, however this approach cannot reliably distinguish between viral strains or infectious from non-infectious viral particles (Dancho et al., 2012). Tissue culture and EM both require expensive equipment and skilled staff, hence are rarely applied for routine examinations. However, early studies investigating the recovery of enteric viruses from sediment usually applied tissue culture for viral enumeration. In order to detect and quantify sediment-associated viruses using tissue culture or EM, viral particles are eluted from sediment and re-concentrated to reduce sample volume. As shown in Table 7A, the usefulness of different approaches has been evaluated, and recoveries exhibited high variations depending on methodology and sediment/virus type. The best recoveries (>60%) were achieved with the use of casein or beef extract solution as an eluent, followed by polyethylene glycol (PEG) precipitation (Johnson et al., 1984; Lewis et al., 1985).

**Table 7:** **Enteric virus recoveries from sediment**.

**Target virus**	**Sediment**	**Elution**	**Concentration**	**Quantitation**	**Mean recovery or range**	**References**
**A INDIRECT EXTRACTION: VIRUS ELUTION—CONCENTRATION**						
Poliovirus 1	Marine 500 g	0.25 M glycine, 0.05 M EDTA, pH 11	0.06 M AlCl_3_, pH 3.5	Culturing	50%	Gerba et al., 1977b
	Marine 10 g	6% beef extract, pH 9	8% PEG6000	Culturing	6.3–55.8%	Lewis et al., 1985
	Marine 10 g	4 M urea, 0.05 M lysine, pH 9	0.005 M AlCl_3_, pH 7	Culturing	22%	Bitton et al., 1982
		3% beef extract	pH 3.5–4.5		8%	
		1% purified casein	pH 3.5–4.5		14%	
	Estuarine, sewage contaminated 10 g	0.25 M glycine	None	Culturing	2.2–3.5%	Tsai et al., 1983
		0.5% skimmed milk			0.5–2.7%	
		0.5% isoelectric casein			58.8%	
		3–10% beef extract (paste)			4.0–9.3%	
		3–10% beef extract (powder)			32.7–40.0%	
		4% nutrient broth			40–53%	
	Estuarine 10–50 mL	3% beef extract, 2 M NaNO_3_, pH 5.5	2 M (NH_4_)_2_SO_4_, 0.01 Cat-Floc T pH 3.5	Culturing	39–44%	Wait and Sobsey, 1983
		3% beef extract, 0.25 M glycine, pH 10.50.25 M glycine, 0.05 M EDTA, pH 11	0.06 M AlCl_3_, pH 3.5		9.7–18%0–0.1%	
	Freshwater10 g	6% beef extract, pH 9	8% PEG6000	Culturing	15.8–76.8%	Lewis et al., 1985
	Freshwater4 types with different sand/silt / clay ratio	4% nutrient broth, pH 7.5	None	Culturing	5.3–10.4%	Johnson et al., 1984
		4% nutrient broth, pH 9			2.0–32.5%	
		5% beef extract, pH 9			0.6–48.9%	
		0.5% isoelectric casein, pH9			0.3–65.3%	
		0.5% isoelectric casein, 1% crude lecithin, pH9			7.0–38.6%	
		0.5% isoelectric casein, 3% crude lecithin, pH9			1.0–25.4%	
		0.5% isoelectric casein, 1% semi-purified lecithin, pH9			0.3–47.9%	
		0.5% isoelectric casein, 3% semi-purified lecithin, pH9			2.6–75.1%	
		0.5% isoelectric casein, 1% egg lecithin, pH9			0.3–56.4%	
		0.5% isoelectric casein, 3% egg lecithin, pH9			0.6–102.6%	
	Silty freshwater 5 g	0.25 M glycine-NaOH, 0.05 M EDTA	0.06 M AlCl_3_, pH 3.5	qRT-PCR	1.8%	Miura et al., 2011
		0.25 M glycine-NaOH, 0.05 M EDTA	0.1 M MgCl2		5.4%	
		0.1% Laureth-12, 0.01 M Tris, 1 mM EDTA, 0.015% Antifoam Y-30, pH 7.2	0.1 M MgCl2		0.61%	
		0.25 M glycine-NaOH, 0.05 M EDTA	16% PEG6000, 4.7% NaCl		0%	
		0.1% Laureth-12, 0.01 M Tris, 1 mM EDTA, 0.015% Antifoam Y-30, pH 7.2	16% PEG6000, 4.7% NaCl		0.18%	
	Sandy freshwater10 g	4 M urea, 0.05 M lysine, pH 9	0.005 M AlCl_3_, pH 7	Culturing	39%	Bitton et al., 1982
		3% beef extract, pH 9	pH 3.5–4.5		51%	
		1% purified casein, 0.1% Tween 80	pH 3.5–4.5		59%	
		1 M trychloroacetate, 1 M glycine	pH 3.5–4.5		23%	
Coxsachievirus B1	Freshwater4 types with different sand/silt/ clay ratio	0.5% isoelectric casein, 1% crude lecithin, pH9	None	Culturing	12.6–37.8%	Johnson et al., 1984
		0.5% isoelectric casein, 3% crude lecithin, pH9			21.8–80.9%	
		0.5% isoelectric casein, 1% semi-purified lecithin, pH9			48.4–61.6%	
		0.5% isoelectric casein, 3% semi-purified lecithin, pH9			54.4–91.5%	
		0.5% isoelectric casein, 1% egg lecithin, pH9			38.6–69.0%	
		0.5% isoelectric casein, 3% egg lecithin, pH9			41.9–73.4%	
Coxsachievirus B3	Estuarine, sewage contaminated 10 g	0.25 M glycine	None	Culturing	4.3–7.7%	Tsai et al., 1983
		0.5% skim milk			8.3–9.2%	
		0.5% isoelectric casein			18.9%	
		3–10% beef extract (powder)			13.0–29.0%	
		4% nutrient broth			15.4–25.9%	
Echovirus 1	Estuarine 10–50 mL	3% beef extract, 2 M NaNO_3_, pH 5.5	2 M (NH_4_)_2_SO_4_, 0.01 Cat-Floc T	Culturing	16–43%	Wait and Sobsey, 1983
		3% beef extract, 0.25 M glycine, pH 10.5	pH 3.5		2.6–4.4%	
		0.25 M glycine, 0.05 M EDTA, pH 11	0.06 M AlCl_3_, pH 3.5		0.1–0.5%	
Echovirus 11	Freshwater4 types with different sand/silt/ clay ratio	0.5% isoelectric casein, 1% crude lecithin, pH9	None	Culturing	5.6–42.4%	Johnson et al., 1984
		0.5% isoelectric casein, 3% crude lecithin, pH9			33.2–94.7%	
		0.5% isoelectric casein, 1% semi-purified lecithin, pH9			31.6–81.9%	
		0.5% isoelectric casein, 3% semi-purified lecithin, pH9			56.8–78.5%	
		0.5% isoelectric casein, 1% egg lecithin, pH9			66.7–138.4%	
		0.5% isoelectric casein, 3% egg lecithin, pH9			43.3–50.4%	
Hepatitis A virus	Freshwater	3% beef extract, 2 M NaNO_3_, pH 5.5	15% PEG6000	Culturing	70%	Lewis and Metcalf, 1988
Simian rotavirus 11	Estuarine10–50 mL	3% beef extract, 2 M NaNO_3_, pH 5.5	2 M (NH_4_)_2_SO_4_, 0.01 Cat-Floc T	Culturing	23%	Wait and Sobsey, 1983
		3% beef extract, 0.25 M glycine, pH 10.5	pH 3.5		0%	
		0.25 M glycine, 0.05 M EDTA, pH 11	0.06 M AlCl_3_, pH 3.5		0%	
Rotavirus WA	Freshwater	3% beef extract, 2 M NaNO_3_, pH 5.5	15% PEG6000	Culturing	70%	Lewis and Metcalf, 1988
**Target virus**	**Sediment**	**Lysis**	**Extraction/concentration**	**Purification**	**Mean recovery or range**	**References**
**B DIRECT EXTRACTION OF VIRAL NUCLEIC ACIDS FOLLOWED BY QRT-PCR**						
Poliovirus 1	Silty freshwater 5 g	0.5 N Tris (pH 8), 0.1 M NaCl, 2% SDS, 8 mg skim milk/g sediment	Phenol:chloroform:isoamyl alcohol + isopropanol precipitation	DEAE cellulose column	0.09%	Miura et al., 2011
		0.5N Tris (pH 8), 0.1 M NaCl, 2% SDS, 8 mg skim milk/g sediment	Phenol:chloroform:isoamyl alcohol + isopropanol precipitation	Oligo(dT) labeled magnetic beads	0.77%	
		0.1 M EDTA, 0.5 N Tris (pH 8), 0.1 M NaCl, 2% SDS, 8 mg skim milk/g sediment	Phenol:chloroform:isoamyl alcohol + isopropanol precipitation	Oligo(dT) labeled magnetic beads	11%	
		TRIzol reagent	Centrifugation	Oligo(dT) labeled magnetic beads	0.10%	

The ELISA approach involves binding of viral antigens to specific antibodies that are subsequently quantified by adding an enzymatic substrate that produces color changes when bound. This technique has been applied in environmental studies (Fu et al., 1989; Park et al., 2010), and results correlate well with tissue culture findings (Nasser et al., 1995). For many enteric viruses, commercial ELISA kits are available allowing rapid detection, however, the assay may detect degraded viral capsid along with infectious particles. The usefulness of ELISA for sediment samples has not been investigated. The most frequently used methods for viral enumeration are qPCR and RT-qPCR which quantify a small segment of the viral genome of DNA and RNA viruses, respectively. These assays are rapid, sensitive, suitable for all virus types, and can be selective for individual strains (Girones et al., 2010). However standard PCR approaches do not provide any information on the integrity and infectivity of the target virus. When (RT-)qPCR is used, the elution of viral particles is not necessary as nucleic acids can be extracted directly from sediment. Recoveries of viral RNA from sediments range from 0.09 to 11% for direct extraction and RT-qPCR, with improved extraction efficiency when applying indirect elution-concentration approaches (Table 7). There are numerous reports of inhibition of PCR assays by organic matter (e.g., humic acids) often found in environmental samples (Meschke and Sobsey, 1998; Rock et al., 2010) and extraction and enumeration methods strongly influence estimates of viral abundance in sediments (Williamson et al., 2013), which can greatly influence attributed risk in pathogenic strains (Petterson et al., 2015). Recently, methods have been applied to overcome this; for example, Carreira et al. (2015) found that a combination of EDTA in addition to probe sonication and enzymatic pre-treatments resulted in 4.5 fold increase in viral recovery from sediments. Miura et al. (2011) found that a direct extraction method utilizing SDS, EDTA coupled with phenol-chloroform-isoamyl alcohol resulted in an 11% recovery of poliovirus 1 (Table 7). Commercial kits for environmental applications are also available and used to extract viral nucleic acids from various matrixes including biosolids (Ikner et al., 2012), however, their efficiency for sediment has not been evaluated. Comparison of viral abundance in sediments (enterovirus 10^2^.g^−1^) to the titre which is shed from infected individuals (10^5^–10^8^.g^−1^) and the high adsorption efficiencies measured in *vitro* (Table 5) suggests dilution, dispersal, and/or high inactivation in sediments (Melnick and Rennick, 1980; Miura et al., 2011).

Determining viral infectivity is a particular challenge in sediments. Most recently, integrated cell culture (ICC) qPCR/RT-qPCR approaches have been developed (Greening et al., 2002; Fongaro et al., 2013; Ogorzaly et al., 2013). During the assay, cultured viruses are enumerated using qPCR or RT-qPCR, which are more sensitive than microscopy and less affected by viral aggregation. This combined approach allows the accurate quantification of infectious viral particles for strains that can be cultured *in vitro* within days (Ogorzaly et al., 2013). Viral recoveries may be improved by the combination of traditional indirect extraction followed by ICC-RT-qPCR (Fongaro et al., 2013). An initial assessment of direct nucleic acid extraction followed by RT-qPCR may be useful for rapid evaluation. Also sediments are difficult to isolate and purify viruses without leaking other compounds which also affect quantification. Research on both improving viral recoveries and role of sediment on the persistence of human pathogenic viruses in the environment could further inform modeling viral pathogens, environmental epidemiology and improve risk assessment.

## Sediments as a sink/source of fecal bacteria and viruses?

Sediments may accumulate enteric bacteria and viruses and release them back in to the water under specific conditions. Therefore, quantifying the mass balance of fecally derived organisms in an estuary is not a simple task. Inputs of bacteria and viruses will be different for each estuary, depending on the surrounding land use, water use, and hydrological processes such as rainfall and tides. Spatial variation within the estuary itself can also confound the issue (Quilliam et al., 2011a; Perkins et al., 2014), as can the seasonal prevalence of bacteria and viruses (Ishii et al., 2006; He and He, 2008; Siem-Jorgensen et al., 2008). Characterization of individual estuaries is underway (Stapleton et al., 2007; Ouattara et al., 2011; Huang et al., 2015) and data from these surveys are being used in models that monitor the fluxes of FIOs, with the primary aim of predicting beach closures due to poor water quality (Stapleton et al., 2007; He and He, 2008; de Brauwere et al., 2014). The sources of fecally derived bacteria and viruses in the typical mixed estuary include wastewater, agricultural runoff, persistent populations, *in situ* growth and infrequent deposition events such as animal feces. The vast majority of fecally derived inputs from agriculture are due to livestock farming, although run-off from arable farming may also contribute to the bacterial/viral loading (Cox et al., 2005). Understanding viral pathogen persistence in wastewater treatment works and whether these viruses persist in sediments is in its infancy (Miura et al., 2011; Kitajima et al., 2014). In contrast bacterial persistence has been studied in detail. For example, Ouattara et al. (2011) reported that wastewater inputs of *E. coli* and intestinal enterococci were 35 and 15 times higher, respectively, than non-point source inputs in the Scheldt Estuary. Weather can also impact the relative contributions of agricultural (diffuse) and wastewater (point source) inputs (Stapleton et al., 2007) further complicating the understanding of pathogen behavior.

### Deposition and retention of fecal bacteria in sediments

Settling and deposition of FIOs and pathogens in sediments is a complex process. Laboratory based estimates of settling velocities are 1.17 and 2.4 m/d for small (0.45–10 μm) and large (>10 μm) particles respectively (Auer and Niehaus, 1993). From a modeling perspective, an approximate deposition rate is taken, although reports vary with reported ranges from 2.6 to 25 md^−1^ (Jamieson, R. et al., 2005). For a review see Pachepsky and Shelton (2011). The settling rate in the field, however, is likely to be lower than these estimates and vary depending on other factors such as turbulence due to waves, wind and tides (Malham et al., 2014). Jamieson, R. et al. (2005) suggested that high bed shear stress limits the exchange between sediments and water column, although in contrast Drummond et al. (2014a) found that deposition of both *E. coli* and inert fluorescent beads occur rapidly, with 74% of the *E. coli* in the top 3 cm. Biofilms, vegetation, organic debris and flocs are likely to reduce the deposition and exchange of FIOs and pathogens to the sediment bed (Arnon et al., 2010; Drummond et al., 2014a,b). Arnon et al. (2010) found that the greater flow velocity and sediment particle size increases the mass transfer of particulate and soluble tracers to the sediments and biofilm and that particles preferentially deposit in biofilms as opposed to underlying sediment. Soluble matter is subject to advective and diffuse mass transport between the water column and bed, particulate matter including FIOs are subject to transport, sedimentation, and filtration (Ren and Packman, 2002; Arnon et al., 2010). The dynamic exchange between deposition and resuspension has received increased attention recently.

### Release and resuspension of bacteria from sediments

During base flow and in the absence of turbulence, sediment-bound bacteria are unlikely to contribute to the bacterial pathogen abundance in the water column (Pachepsky and Shelton, 2011). Turbulence generated during peak flow results in mixing, an increase in oxygenation, bubble generation, and shear stress, which increases detachment rates from sediment and is dependent on bacterial shape and strain, and biofilm cohesive strength (Gomez-Suarez et al., 2001; Young, 2006; Lemos et al., 2014; Figure 1D). The release/resuspension of bacteria from biofilms within sediments is dependent on the combination of physicochemical forcing (Walter et al., 2013) and biotic factors, such as grazing and quorum sensing (Costerton et al., 1995; Kim et al., 2016) which could impact particulate loading to the water column (Figure 1E).

The release of *E. coli* from estuarine silts has been linked to rapid decreases in salinity of the water, which can occur in estuarine environments (Weiss, 1951). However, in freshwater systems, the number of *E. coli* released in successive events are limited to the deposited/proliferated bacteria between events (Shelton et al., 2014) and the sediment depth which is subject to scour (Harvey et al., 2012). The bacterial abundance increases in the water column on the rising curve of the storm hydrograph, due to particulate resuspension under periods of high turbulence (Howlett et al., 2015) often with a delay between the peak in riverflow and the peak in bacterial abundance (Jamieson R. C. et al., 2005; Jamieson R. et al., 2005; Henson et al., 2007). Controlled water release from a reservoir to a stream accounted for a 1–2 log increase in *E. coli* in the water column, but is dependent on the abundance in the sediment (Drummond et al., 2014a). Similarly, for viruses, desorption of viruses from clay particles can be attributed to reductions in salinity and the addition of organic matter due to rainfall or tides (Gerba and Schaiberger, 1975). In saline environments cations such as Ca^2+^ form bridges to stabilize bacteria binding to the sediment and also decreases the electrostatic repulsion during the initial stages of adsorption (van Loosdrecht et al., 1989; de Brouwer et al., 2002; Kierek and Watnick, 2003). The input of freshwater into estuarine systems could reduce the efficacy of these bridges, releasing pathogenic bacteria such as *V. cholerae* from sediment into the water column (Kierek and Watnick, 2003). Guizien et al. (2014) found that bacterial numbers decreased due to grazing and that viral titre in the water column did not significantly increase due to re-settlement of virus-clay complex, suggesting a complex story governing resuspension of enteric microorganisms. The risk associated with the sediment of enteric microorganisms depends on concentration, the ease with which bacteria resuspend or release back into the water column, and the frequency with which this will occur (Cox et al., 2005). Understanding viral resuspension in the environment is reliant on development of suitable methodology to enumerate viruses with accuracy and precision in a reproducible manner.

## Outlook

Traditional molecular approaches are useful for absolute quantification of target organisms (e.g., *E. coli, Salmonella* spp, *Enterococcus* spp). However, advances in high throughput sequencing (HTS) have been applied to monitor fecal pollution, on a variety of different environmental matrices including wastewater, drinking water, riverine/coastal waters and ground water (Tan et al., 2015). For a more comprehensive review of HTS for assessing water quality see Tan et al. (2015). Targeted sequencing of 16S rRNA for bacteria and 18S rRNA for eukaryote small sub-unit rRNA permits an estimate of diversity and abundance (Henry et al., 2016), although the resolution of the 18S RNA gene as a phylogenetic marker us variable amongst taxa and is often not suitable for the resolution of FIOs belonging to the enterobacteriaceae for example. However, coupled sequencing and flow cytometry approaches can be used for more accurate taxon quantification (Props et al., 2016), although a different quantitative technology would be required for sediments. Bacterial diversity can be readily established in sediments using HTS; however rare sequences (e.g., pathogens) may evade detection, which is dependent on sequencing depth. Therefore, quantitative methods (e.g., qPCR, RT-qPCR) in sediments, remain a critical approach for inferring quantitative change associated with relative abundance HTS datasets.

Host associated genetic markers from bacterial groups such as *Bacteroidales* have been identified in sediments, which provides useful information for source apportionment (Tan et al., 2015). Genetic fingerprints of 16S rRNA gene or metagenome sequencing can reveal similarities between source (outfall, runoff etc.) and sink (beach sands and sediments) through community analysis (Ervin et al., 2014; Neave et al., 2014). Neave et al. (2014) confirmed the importance of local pollution sources of fecal species, in determining the fecally derived component of sediment associated microbial communities, which have a significant impact on beach sediment quality (e.g., Vignaroli et al., 2013). Sediments provide natural areas of high microbial density, which is of particular concern considered the elevated persistence and accumulation of antimicrobial resistance (AMR). Port et al. (2014) estimated the potential for gene transfer and pathogenicity potential from sediment fecal bacteria from pyrosequencing datasets of sediments. This is pertinent for developing suitable baseline AMR/pathogenicity levels used to inform policy makers on fecally derived hazardous microorganisms/pathogens in sediments. The structuring effect of physiochemical variables such as salinity and sediment porosity on bacterial communities is unsurprising (Hamdan et al., 2013); from a pollution perspective, chemical contamination appears to drive bacterial community structure at least at a local level (Staley et al., 2014), although FIOs appear strongly source dependent.

HTS data has provided novel insights into the dynamics of sediment associated enteric viruses (Paez-Espino et al., 2016). Predictions of viral relative abundance and potential pathogenicity genes can be undertaken with HTS of sediments (Yoshida et al., 2013). However, viral enrichment is often required for the detection of pathogenic components of the virome. Concentration through tangential ultraflow filtration has been applied on dispersed sludges. Following this, immunoprecipitation through antibodies, affinity capture has been applied to isolate pathogenic polioviruses, followed by deep HTS (Furtak et al., 2016), although to our knowledge this approach has yet to be applied for sediments. From an environmental quality perspective, understanding the physiochemical drivers governing the mainitence of the abundance and persistence of viruses and bacterial pathogens in sediments is of principal concern for regulators and requires more attention.

## Conclusions

It is anticipated that enteric microorganisms in sediments will continue to be of significant interest for the foreseeable future. It is unlikely that environmental legislation will be widened to cover FIOs/pathogens in sediments; however, understanding the fate of enteric and pathogenic viruses in the environment, including sediments, will be important for implementing potential viral standards. The influence of wastewater treatment works on viral abundance and infectivity, VBNC bacteria, and the role of particulate matter and sediments on source/sinks of these organisms is still unclear. Furthermore, a lack of standardized effective methods for enumerating both VBNC and viruses from environmental matrices (including sediments) has hampered research in these areas. Significant headway on applying correction factors for viral extractions using internal standards has shed light on the problem of poor extraction efficacy and inhibition of molecular methods. However, these workaround methods have yet to be applied to sediment and do not address the fundamental quantification problems. Environmental monitoring should now apply a holistic approach using novel technologies such as “lab on chip” and structural integrity methods which may improve on site diagnostics and should include tests for viral infectivity where this is lacking, all of which are currently not applied to sediments. Further studies are also required to close the loop between conventional plate count and qPCR based methods in sediments particularly regarding the role of VBNC bacteria (if any) on bathing water quality and human/environmental health. An improved process-level understanding is required to consider if FIOs and pathogens from different sources (diffuse or point source derived or from human or animal) have different VBNC or resuscitation potentials. Complete life cycle analysis from terrestrial, fluvial and coastal zones is also required to fully understand the role of sediments in viral transport and infectivity persistence in the environment. Studies which model pathogenic viral abundance to observed FIO numbers have been successfully applied to water and applicability in sediments requires increased attention. Finally, increased acquisition of physiochemical data in addition to routine biological samples will improve our understanding of the fate of viruses, VBNCs and FIOs in sediments and enable the development of suitable environmental risk assessment for microbiological risk of sediments to human health. In conclusion, our poor process-level understanding of viral/bacterial-sediment interactions combined with methodological challenges is limiting accurate source apportionment and quantitative microbial risk assessment for pathogenic organisms associated with sediments in aquatic environments.

## Author contributions

FH, CG, and KF wrote the manuscript and undertook data analysis. AA, VJ, SM, JM, DJ, and BC provided technical support and reviewed the manuscript. FH, SM, KF, JM and DJ prepared manuscript for submission. All authors have approved the final version to be published. All authors had substantial contributions to the conception and design of the work.

### Conflict of interest statement

The authors declare that the research was conducted in the absence of any commercial or financial relationships that could be construed as a potential conflict of interest. The authors were commissioned and funded by United Kingdom Water Industry Research Limited to carry out a revised version of this research, which was reported as chapter presented within a technical report. The funding agency did not influence the content of this review.

## Figures and Tables

**Table:** 

Culturability	The ability of bacteria to form colonies on agar microbiology plates.
Viable but non-culturable	Bacteria which can no longer form colonies on agar plates, but are still metabolically active and remain viable.
Viable	Refers to all living bacteria
